# CNS Involvement in AML Patient Treated with 5-Azacytidine

**DOI:** 10.1155/2014/937203

**Published:** 2014-08-14

**Authors:** Diamantina Vasilatou, Sotirios Papageorgiou, Efthymia Bazani, Athina Prasouli, Christina Economopoulou, Christoforos Roumpakis, Petros Karakitsos, George Dimitriadis, Vasiliki Pappa

**Affiliations:** ^1^Second Department of Internal Medicine and Research Institute, Athens University Medical School, Attikon University General Hospital, 1 Rimini Street, 12462 Athens, Greece; ^2^Iatropolis Magnetic Tomography SA, 15231 Athens, Greece; ^3^Department of Cytopathology, Medical School Attikon University Hospital, University of Athens, 12462 Athens, Greece

## Abstract

Central nervous system (CNS) involvement in acute myeloid leukemia (AML) is a rare complication of the disease and is associated with poor prognosis. Sometimes the clinical presentation can be unspecific and the diagnosis can be very challenging. Here we report a case of CNS infiltration in a patient suffering from AML who presented with normal complete blood count and altered mental status.

## 1. Introduction

Infiltration of CNS in patients with AML is a rare complication and is associated with low rates of complete response and poor prognosis. Several studies highlight the risk factors for AML and stress the importance of recognizing the high risk patients. Here we report a case of an AML patient whose disease relapsed with blasts in the CSF without any sign of relapse in peripheral blood.

## 2. Case Presentation

A 75-year old man with a history of Parkinson disease treated with levodopa and benzerazide was diagnosed with AML (M5 according to FAB classification) and pentasomy 8 {49XY,+8+8+8[21], 46XY[4]}. Due to his age and comorbidity, the patient was treated with one cycle of the combination of idarubicin 12 mg/m^2^ (d1 and 2) and cytarabine 100 mg/m^2^ (d1–5) and achieved complete remission (CR) that was confirmed by bone marrow aspiration and karyotype. However, due to his poor performance status the patient was not eligible for another course of chemotherapy, so he was treated with 6 cycles of azacitidine and remained in CR.

Three weeks after the last cycle of azacitidine, the patient was admitted with an altered level of consciousness. On his admission, the patient presented with impaired mental functions (expressed as impaired memory, cognition, etc.), GCS (Glasgow coma scale) 8, extrapyramidal symptoms, and primitive reflexes such as grasp sign and ataxic gait. In addition, he exhibited dysarthria and impaired left light reflex.

The laboratory tests were unremarkable. Of note, the complete blood count (CBC) on his admission was as follows: white blood cells (WBC) 5520/*μ*L (78.8% neutrophils and 12.9% lymphocytes, 7.8% monocytes and 0.5% eosinophils), hemoglobulin (Hb) 13.6 g/dL, and platelets (PLTs) 120000/*μ*L.

**Figure 1:**
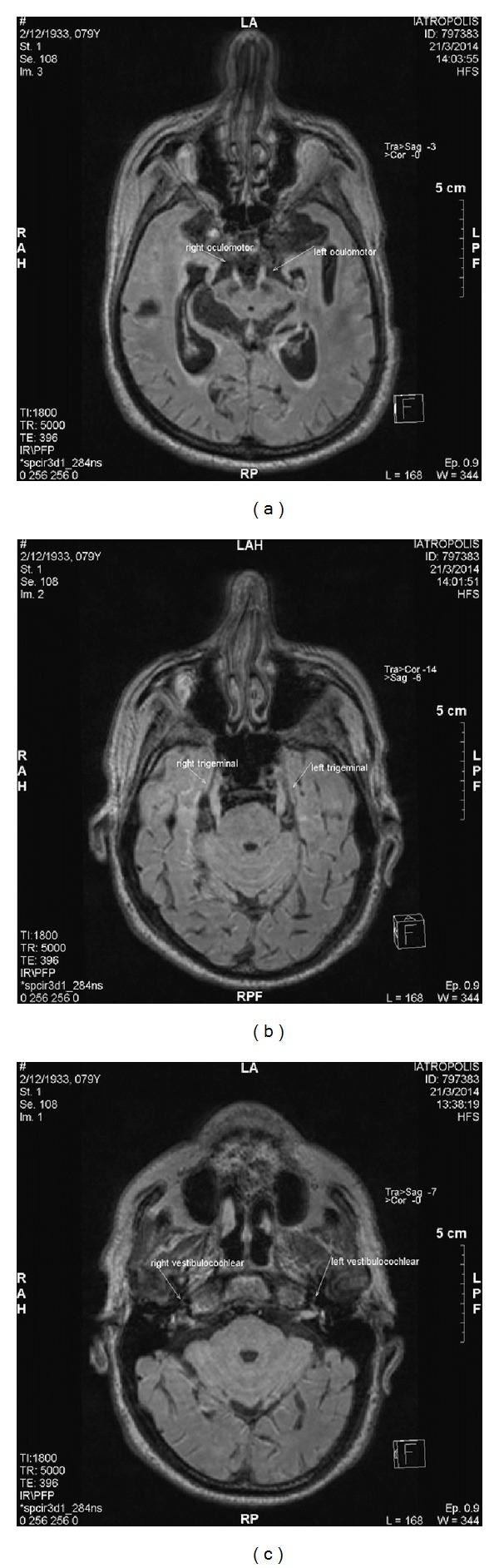
The MRI of the brain showed enhancement of the left trigeminal (a) and both oculomotor (b) and vestibulocochlear nerves (c) and signs of leptomeningeal disease.

The brain CT scan revealed two lesions 1.2 and 1.1 cm on the meninges of the frontal and occipital lobe of right hemisphere and no sign of brain edema. The patient underwent an MRI of the brain that showed enhancement of both trigeminal (especially the left) and both oculomotor and vestibulocochlear nerves and abnormal meningeal enhancement in flair sequence (Figures 1(a)–1(c)). A lumbar puncture was performed and the cerebrospinal fluid (CSF) analysis revealed 390 cells/mm^3^ (20% neutrophils and 80% lymphocytes) and 20 red blood cells (RBC)/mm^3^. The gram strain was negative and so was the Cryptococcus neoformans antigen tested by the Indian ink method. In addition, CSF glucose was 62 mg/dL (with blood glucose: 115 mg/dL), LDH was 277 U/L, total proteins were 325.3 mg/dL (normal values 15–45 mg/dL) and albumin was 268.3 mg/dL.

**Figure 2:**
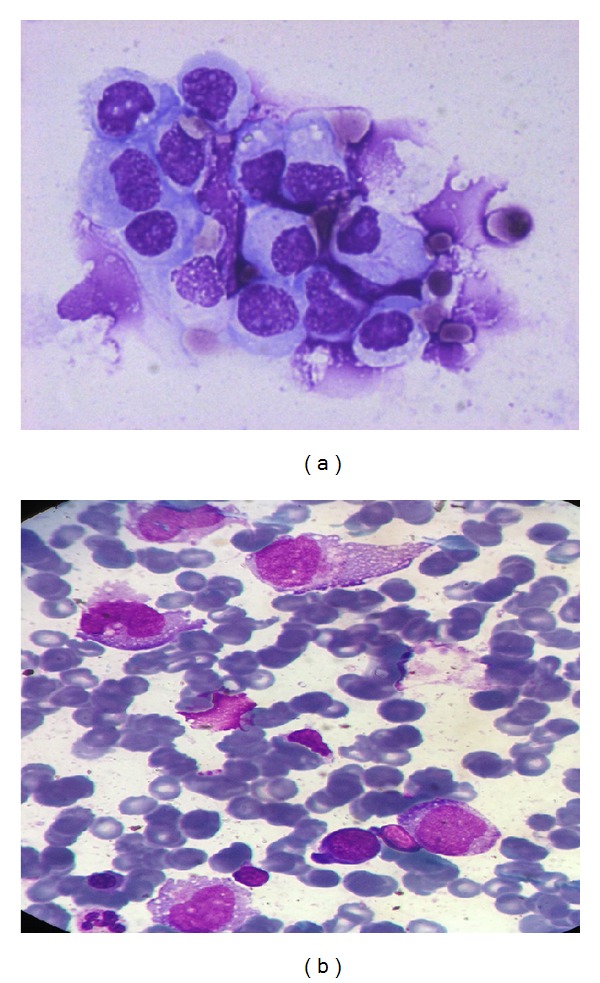
(a) CSF was infiltrated by immature blood cells with characteristics of monoblasts. (b) The same cells were detected in the bone marrow, confirming the diagnosis of AML relapse in bone marrow and CNS.

Waiting for the cytology cultures of the CSF and considering the severity of the patients' clinical condition he was treated with antibiotic, antiviral, and antituberculosis drugs due to the suspicion of infectious meningitis, without improvement. 24 hours later, the cytology test revealed presence of blast cells in the CSF, establishing the diagnosis of CNS infiltration (Figure 2(a)). Bone marrow aspiration confirmed the disease relapse (Figure 2(b)). However, no blasts were isolated from the peripheral blood. The PCR-TB and CSF cultures were completed a few days later and were negative.

After the diagnosis of CNS infiltration the patient received corticosteroids which shortly improved his level of consciousness. Antibiotic, antiviral, and antituberculosis treatments were discontinued. Unfortunately, several days later his clinical condition was complicated again with urinary tract infection by a multiresistant strain of Acinetobacter baumannii. The patient died two weeks later from septic shock.

## 3. Discussion

The exact incidence of CNS involvement in patients suffering from AML is unknown. However, it is less common than CNS infiltration in adults and children with ALL [1, 2]. Its estimated incidence is below 5% at diagnosis, so lumbar puncture is not a routine diagnostic procedure in patients without CNS symptoms [3]. It seems that the use of high dose cytarabine as consolidation in AML treatment has reduced the incidence of CNS infiltration because it penetrates into the CNS (2014, uptodate). Our patient received cytarabine 100 mg/m^2^ and idarubicin 12 mg/m^2^ as induction chemotherapy. There are several phases I and II studies suggesting that sequential use of azacitidine and lenalidomide or gemtuzumab ozogamicin alone is promising agents for elderly with AML [4, 5] but the results need to be validated with phases III and IV studies.

The pathogenesis of CNS leukemia is also under investigation. Several mechanisms have been proposed, like extension from the bone marrow or bony lesions of the skull, contamination of the CSF via the choroid plexus, invasion of the cerebral parenchyma through brain capillaries, hemorrhage into the CNS, and so forth [6]. Since our patient had neither brain hemorrhage nor bony lesion of the skull, it seems that the most possible ways of CSF contamination are extension from the bone marrow of the skull or invasion from brain capillaries.

Risk factors for CNS involvement in AML patients are inv (16) or chromosome 11 abnormalities, trisomy 8, hyperleukocytosis, high percentage peripheral blasts, elevated lactate dehydrogenase (LDH), prominent monocytic component, and acute promyelocytic leukemia (APL) in systemic relapse [6, 7]. As mentioned above, our patient suffered from acute monocytic leukemia with abnormalities of chromosome 8 (pentasomy 8). As a result, the patient belonged to a high risk group for CNS involvement. Furthermore, the expression of CD56 on the leukemia cells has been associated with CNS disease. CD56, also called neural cell adhesion molecule, is a binding glycoprotein normally expressed on the surface of neurons, glia, skeletal muscle, and natural killer cells. Interestingly, CD56 expression is associated with high rates of extramedullary disease, including CNS, low CR rate, and poor overall survival [8, 9]. Flow cytometry of bone marrow at diagnosis revealed that our patient's blast cells were positive for the CD56 molecule.

Clinical presentation of CNS involvement in AML may be indolent. However, patients with symptoms of increased endocranial pressure such as mental changes and headache, cranial nerve palsies, CNS hemorrhage, symptoms of spinal cord compression, and visual changes should be checked for CNS infiltration with lumbar puncture and radiology imaging studies. Most patients exhibit moderate elevation in protein and moderate decrease in glucose, which was true for our patient's CSF (2014, up-to-date). CT and MRI can exclude hemorrhage, stroke, and brain tumor. In addition, in case of cranial nerve palsies and negative CSF, MRI can prove very helpful in recognizing signs of CNS infiltration. In our case, both MRI and CSF examination were indicative of CNS infiltration.

Interestingly, the disease relapse of our patient was first detected in CSF and then in the bone marrow. No blast cells were detected in peripheral blood and his CBC remained normal during his hospitalization. A case series of Cheng et al. revealed nine patients with AML or ALL who presented with symptoms from CNS. Lumbar puncture was performed and blasts were found in the CSF. No blasts were present in peripheral blood while bone marrow aspiration confirmed the diagnosis [10]. These data support the role of CSF examination in the diagnosis of AML relapse. Finally, it is not clear whether azacitidine can cross the blood brain barrier [11] so it can be hypothesized that the treatment with azacitidine can gain control of the disease in the bone marrow but cannot eliminate the possibility of CNS infiltration. However, there are no data to enlighten the role of azacitidine in CNS infiltration of AML.

In conclusion, CNS involvement in AML course is uncommon, its pathogenesis is still under investigation and its clinical presentation can be very indolent. However, there are data suggesting that CNS infiltration with acute myeloid leukemia is indicative of poor prognosis [9], which means that early diagnosis and treatment are more than necessary.
